# HIV risk profile and prevention needs of individuals seeking monkeypox (mpox) vaccination in an urban clinic in the U.S.: a brief report

**DOI:** 10.1186/s12879-023-08075-5

**Published:** 2023-03-08

**Authors:** Onyema Ogbuagu, Manas Sharma, Grace Igiraneza, Laurie Andrews, Jessica Tuan, Lydia A. Barakat

**Affiliations:** 1 Section of Infectious Diseases, Yale School of Medicine, 135 College St., Suite 323, New Haven, CT 06510 USA; 2 Section of Infectious Diseases, Yale Department of Medicine, Yale School of Medicine, Yale University School of Medicine, 333 Cedar Street, PO Box 208022, New Haven, CT 06510 USA; 3 Yale AIDS Program, 135 College St., Suite 323, New Haven, CT 06510 USA; 4 Yale AIDS Program, Section of Infectious Diseases, Yale School of Medicine, 135 College St., Suite 323, New Haven, CT 06510 USA

**Keywords:** Monkeypox, HIV infection, Pre-exposure prophylaxis, PrEP, Vaccination

## Abstract

**Background:**

Our study survey assessed HIV risk profile and pre-exposure prophylaxis (PrEP) use among HIV-negative individuals seeking mpox vaccination, elucidating HIV prevention gaps and opportunities.

**Methods:**

Anonymous cross-sectional surveys were self-administered at an urban academic center clinic in New Haven, CT, U.S. (August 18–November 18, 2022). Inclusion criteria included adults presenting for mpox vaccination who consented to the study. The study assessed STI risk (sexual practices, STI history, substance use). For HIV-negative participants, PrEP knowledge, attitudes, and preferences were assessed.

**Results:**

Eighty-one of 210 individuals approached completed surveys (survey acceptance and completion rate 38.6%). Majority were cisgender-male (76/81; 93.8%), Caucasian (48/79; 60.8%), with median age 28 years (IQR-15). Nine of 81 (11.5%) self-reported HIV-positivity. Median sexual partner number (6 months prior) was 4 (IQR-5.8). Majority, 89.9% and 75.9%, reported insertive and receptive anal intercourse, respectively. 41% reported lifetime STI history, of whom 12.3% had an STI 6 months prior. Majority (55.8%) used ≥ 1 illicit substance; 87.7% moderate alcohol use. Among HIV-negative respondents, most (95.7%) were aware of PrEP, but only 48.4% used PrEP.

**Conclusion:**

Individuals seeking mpox vaccination engage in behaviors placing them at increased STI risk and would benefit from PrEP assessment.

**Supplementary Information:**

The online version contains supplementary material available at 10.1186/s12879-023-08075-5.

## Background

Monkeypox, now renamed mpox, a zoonosis endemic to Central and West Africa since the 1970’s, and has since become a global pandemic beginning in May 2022 [1]. The monkeypox virus that is spread primarily by close contact with and bodily fluids from infected humans or animals [2], has caused over 82,500 mpox cases globally, likely an underestimate due to under-detection and under-reporting. Unique to this pandemic is an avidity for sexual transmission, occurring disproportionately among gay and bisexual men and close contacts [3, 4].

As monkeypox virus is a close relative of smallpox virus (another orthopoxvirus with which it shares similar virologic characteristics [1]), antiviral treatments and vaccines, which were relatively dormant, have been deployed to address the emerging threat [5, 6].

In addition to promoting uptake of biomedical interventions for mpox, public health measures have focused on education, promoting safe sexual practices, and reducing behaviors that would place individuals at increased risk for HIV (e.g., increased number of sexual partners, condomless anal sex, etc.). This current epidemic is occurring in the context of ongoing recent epidemics like SARS CoV-2 infection/COVID-19 and long-term epidemics such as HIV. In some cases, ongoing epidemics share similar modes of transmission and affect similar populations, a phenomenon occasionally referred to as syndemics [7]. This provides opportunity and occasion to cross-utilize relevant prevention approaches to concurrent epidemics.

As gay and bisexual men were at increased risk for mpox, they were priority groups for mpox vaccination (using the mpox expanded post-exposure prophylaxis PEP++ vaccination strategy). Those eligible for mpox vaccination under PEP++ vaccination strategy included the following: (1) Known contacts to someone with mpox, (2) People aware that a recent sex partner was diagnosed with mpox (within past 14 days), (3) Gay, bisexual, and other men who have sex with men (MSM); and transgender, nonbinary, and gender-diverse people; who have experienced at least of the following (within past 14 days): sex with multiple partners; at a commercial sex venue; or in association with an event, venue, or area where mpox transmission is occurring. As approximately 25% of those at risk of acquiring HIV are utilizing HIV pre-exposure prophylaxis (PrEP) in the U.S. [8], one way to increase reach is to utilize opportunities and venues where individuals source other health services to assess needs and provide additional sexual and reproductive health services as indicated [9].

According to the Connecticut Department of Public Health, in 2019, in New Haven County, there were 3,380 people living with HIV and the rate of people living with HIV was 395 per 100,000. There were greater than 25 new cases of HIV diagnosed in New Haven, Connecticut in 2019. In 2021 in Connecticut, the number of HIV PrEP users was 3234. The rate of PrEP users per 100,000 population in the same year was 106 [10]. PrEP is covered by private and state insurance in Connecticut. Options include oral agents and injectables. To provide contextualization, in a longitudinal study performed of MSM on HIV PrEP in the New Haven, CT and Providence, Rhode Island setting (2015–2017), demonstrated that the median number of sexual partners in the past 3 months was 3, reported illicit drug use was 57%, and 20% had had sex with a known partner with HIV in the past 3 months [11]. With regards to mpox, at the time our study was conducted, there were 144 cases of monkeypox reported in Connecticut.

Thus, our study aimed to assess HIV risk behaviors, knowledge, uptake, and preferences of prevention services among individuals seeking mpox vaccination at an urban, outpatient academic center infectious diseases clinic.

## Methods

The study was conducted at the Nathan Smith Clinic of Yale-New Haven Health academic center in New Haven, CT, U.S. Our clinic was designated by the Connecticut Department of Public Health as a site of mpox vaccination in July 2022. Our study population consisted of individuals seeking vaccines from the greater New Haven, Connecticut area and beyond. From August 18–November 18, 2022, we administered a paper-based survey (based on CDC HIV PrEP risk assessment tool and expanded monkeypox PEP++ criteria), that assessed multiple domains including: (1) Demographics, (2) Sexual history (including orientation, range of sexual practices, number of sexual partners, and STIs), (3) Medical history (including substance use), (4) Knowledge of HIV PrEP, and (5) Preferences for PrEP (administration route). The final question in our survey for those who were PrEP-unaware and who through the questionnaire became aware of PrEP were offered the opportunity to have a PrEP encounter at our clinic.

Eligible participants were adults aged ≥ 18 years who voluntarily and anonymously agreed to complete surveys (offered by receptionist staff to all individuals). Adults with HIV were asked to not complete PrEP-related sections. This study was approved by the Yale Institutional Review Board.

### Statistics

Descriptive statistics were utilized through Microsoft Office Excel software to analyze subject data and report proportions of individuals reporting pre-specified behaviors, practices, and treatment preferences. We analyzed data on PrEP use and preferences for self-reported HIV-negative participants.

## Results

### Demographics and behavioral data

We received 81 completed surveys of 210 individuals approached. Most participants were assigned male at birth (AMAB, 93.8%). 1.2% of respondents reported being assigned female at birth (AFAB); and 1.2% identified as non-binary and non-conforming, respectively. Median age was 28 years. 85% were single. Of 79 respondents, 60.8% were White and 16.4% Black. 15.2% identified as Hispanic ethnicity (Table 1).

**Table 1:** Demographics and characteristics, history of at-risk behaviors, PrEP awareness and preferences of survey subjects

Characteristics	n (%) or median (IQR)	HIV-positive (n = 9)	HIV-negative (n = 69)
Race/ethnicity	79 (100)	9 (100)	70 (100)
Black	13 (16.4)	2 (22.2)	11 (15.7)
White	48 (60.8)	4 (44.4)	44 (62.9)
Asian	14 (17.7)	2 (22.2)	12 (17.1)
Pacific Islander/other	4 (5.1)	0 (0)	4 (5.7)
Mixed race	5 (6.3)	1 (11.1)	4 (5.7)
Hispanic/LatinX	12 (15.2)	1 (11.1)	11 (15.7)
Gender	81 (100)	9 (100)	68 (100)
Cis-Male	76 (93.8)	9 (100)	63 (92.6)
Cis-Female	2 (2.4)	0 (0)	2 (2.9)
Trans-male	1 (1.2)	0 (0)	1 (1.5)
Non-binary	1 (1.2)	0 (0)	1 (1.5)
Non-conforming	1 (1.2)	0 (0)	1 (1.5)
Age (years)	28 (15)	39 (25)	27 (13.3)
Occupation	77 (100)	9 (100)	68 (100)
Healthcare worker	10 (13)	1 (11.1)	10 (14.7)
Other at-risk occupation	6 (7.8)	0 (0)	6 (8.8)
Educational level	80 (100)	9 (100)	69 (100)
Less than high school	0 (0)	0 (0)	0 (0)
Completed high school	19 (23.8)	3 (33.3)	16 (23.2)
College degree	22 (27.5)	1 (11.1)	22 (31.9)
Post-graduate or other advanced degrees	36 (45)	5 (55.6)	30 (43.5)
Annual income	76 (100)	4 (100)	66 (100)
$75K or greater	23 (30.3)	3 (75)	21 (31.8)
$40K–$74K	13 (17.1)	0 (0)	12 (18.2)
$20K–$39K	20 (26.3)	0 (0)	15 (22.7)
< $20K	20 (26.3)	1 (25)	18 (27.3)
Medical insurance	78 (100)	9 (100)	68 (100)
Private insurance	64 (82.1)	5 (55.6)	57 (83.8)
Medicaid	7 (9)	2 (22.2)	5 (7.4)
Medicare	7 (9)	0 (0)	6 (8.8)
None	0 (0)		0 (0)

Behaviors			
Gender of sex partners	81 (100)	9 (100)	69 (100)
Male alone	69 (85.2)	8 (88.9)	58 (84.1)
Female alone	1 (1.2)	0 (0)	1 (1.4)
Both	11 (13.6)	1 (11.1)	10 (14.5)
Number of sexual partners			
Past 6 months	4 (5.8)	4 (3.9)	4 (6)
Past 2 weeks	1 (1)	1 (0)	1 (1)
Intimate behaviors/sexual practices	79 (100)	9 (100)	69 (100)
Hugging	70 (88.0)	5 (55.6)	64 (92.8)
Kissing	72 (91.1)	5 (55.6)	65 (94.2)
Oral intercourse	76 (96.2)	7 (77.8)	69 (100)
Insertive anal intercourse	55 (69.6)	5 (55.6)	48 (69.5)
Receptive anal intercourse	50 (63.3)	4 (44.4)	46 (66.7)
Insertive vaginal intercourse	6 (7.6)	1 (11.1)	5 (7.2)
Condom usage	90% (65.3%)	80% (75%)	90% (61.8%)
HIV status	81 (100)		
Positive	11.50%		
Negative	84.80%		
Unknown	3.70%		
STI history	81 (100)	9 (100)	69 (100)
Ever	33 (40.7)	7 (77.8)	25 (36.2)
Gonorrhea	17 (21)	5 (55.6)	12 (17.4)
Chlamydia	15 (18.5)	2 (22.2)	12 (17.4)
Syphilis	11 (13.6)	5 (55.56)	6 (8.7)
HPV	8 (9.9)	1 (11.1)	7 (10.1)
Genital herpes	1 (1.2)	1 (11.1)	0 (0)
Past 6 months	10 (12.3)	2 (22.22)	8 (9.9)
Gonorrhea	7 (8.6)	2 (22.2)	5 (6.2)
Chlamydia	3 (3.7)	2 (22.2)	2 (2.5)
Syphilis	2 (2.5)	1 (11.1)	2 (2.5)
Trichomoniasis	1 (1.2)	0 (0)	1 (1.2)
HPV	1 (1.2)	1 (11.1)	1 (1.2)
*M. genitalium*	1 (1.2)	0 (0)	1 (1.2)
Genital herpes	0 (0)	0 (0)	0 (0)
Substance use	77 (100)	9 (100)	67 (100)
No	34 (44.2)	3 (33.3)	31 (46.3)
Yes	43 (55.8)	6 (66.7)	36 (53.7)
Marijuana	37 (48.1)	4 (44.4)	32 (47.8)
Poppers or other inhalants	19 (24.7)	3 (33.3)	16 (23.9)
Cocaine	14 (18.2)	2 (22.2)	12 (17.9)
Ketamine	6 (7.8)	2 (22.2)	4 (6)
Methamphetamine	5 (6.5)	3 (33.3)	2 (3)
Mushrooms/psilocybin	3 (3.9)	0 (0)	3 (4.5)
MDMA	1 (1.3)	0 (0)	1 (1.5)
LSD	1 (1.3)	0 (0)	1 (1.5)
PCP	1 (1.3)	0 (0)	1 (1.5)
Heroin	1 (1.3)	0 (0)	1(1.5)
Tobacco	71 (100)	9 (100)	61 (100)
Yes	13 (18.3)	3 (33.3)	10 (13.1)
No	58 (81.7)	6 (66.7)	51 (86.9)
Alcohol	73 (100)	9 (100)	63 (100)
Yes	64 (87.7)	7 (77.8)	56 (88.9)
Moderate	64 (87.7)	7 (100)	56 (88.9)
Heavy	0 (0)	0 (0)	0 (0)
No	9 (12.3)	2 (22.2)	7 (11.1)
Aware of PrEP	79 (100)	9 (100)	69 (100)
Yes	75 (94.9)	8 (88.9)	66 (95.7)
No	4 (5.1)	1 (11.1)	3 (4.3)
Brand awareness	65 (100)	2 (100)	63 (100)
Tenofovir disoproxil fumarate/emtricitabine	43 (65.8)	2 (100)	41 (65.1)
	29 (44.4)	2 (100)	27 (42.9)
Tenofovir alafenamide/emtricitabine	4 (6.3)	0 (0)	4 (6.3)
Cabotegravir			
PrEP usage	69 (100)		69 (100)
Yes	33 (48.1)		33 (48.1)
No	36 (51.9)		36 (51.9)
Brand usage	33 (100)		33 (100)
Tenofovir disoproxil fumarate/emtricitabine	21 (63.6)		21 (63.6)
Tenofovir alafenamide/emtricitabine	11 (33.3)		11 (33.3)
Cabotegravir	1 (3)		1 (3)
Prescription provider	32 (100)		32 (100)
Primary care provider	24 (75)		24 (75)
HIV specialist/clinician	4 (12.5)		4 (12.5)
Other sources	4 (12.5)		4 (12.5)
Adherence	28 (100)		28 (100)
Full (no missed doses)	21 (75)		21 (75)
At least 4 doses per week	2 (7)		2 (7)
Less than 4 doses a week	5 (17.9)		5 (17.9)
Preferred route of administration	40 (100)		40 (100)
Oral agent	30 (75.8)		30 (75.8)
Injectable agent into muscle	5 (13)		5 (13)
Injectable agent into skin	4 (10.1)		4 (10.1)
Implant	1 (4.3)		1 (4.3)
Openness to self-injection	42 (100)		42 (100)
Yes	18 (42.9)		18 (42.9)
No	24 (57.1)		24 (57.1)
Preferred frequency	44 (100)		44 (100)
Daily	7 (15.9)		7 (15.9)
Weekly	11 (25)		11 (25)
Monthly	14 (31.8)		14 (31.8)
Every 2 months	5 (11.2)		5 (11.2)
Every 6 months	8 (18.4)		8 (18.4)
1 year or more	17 (38.6)		17 (38.6)

The percentages reported represent the proportions of respondents who addressed each question

*PrEP* pre-exposure prophylaxis, *STI* sexually transmitted illness, *HPV* Human papillomavirus, *MDMA* 3,4-Methyl enedioxy methamphetamine, *LSD* lysergic acid diethylamide, *PCP* phencyclidine

The gender of sexual partners was only male for 85.2%, while 13.6% reported both, and 1.2% selected only female partners. Median values for number of sexual partners in prior 6 months and 2 weeks were 4 (IQR-5.8) and 1, respectively (IQR-1). 12.3% had partners living with HIV (Table 1). Regarding sexual practices, 96.2% participated in oral sex, 69.6% insertive anal intercourse, 63.3% receptive anal intercourse, and 7.6% insertive vaginal intercourse. Median percentage of condom usage was 90% (IQR-65.3%). Majority (94.9%) reported no known mpox exposure, while 3.8% reported known exposure.

Of respondents, 40.7% had a lifetime STI history (defined as any self-reported STI since birth and without prejudice to etiology) and 12.3% had an STI within 6 months prior; furthermore, 11.5% were self-reported HIV-positive, and 84.8% were HIV-negative (Table 1). Of the self-reported HIV-negative participants, only 34.2% reported a negative HIV test within 3 months prior.

Use of ≥ 1 illicit substance was reported by 55.8% of participants, most commonly marijuana (48.1%). 87.7% reported moderate alcohol use [12] (Table 1).

### HIV PrEP awareness and preferences

Of 79 respondents, 94.9% reported PrEP awareness (Table 1). 65.8% and 6.3% reported knowledge of tenofovir disoproxil fumarate/emtricitabine and cabotegravir, respectively; while 13.3% reported unawareness of any of these medications.

Of 69 HIV-negative individuals, only 48.1% reported PrEP use, with 75% reporting full adherence. 7% of respondents reported dissatisfaction with their current PrEP modality (citing “difficulty” and/or “disliking swallowing pill”). One-third of those with an STI history and other behavioral risks for HIV were not on PrEP. Forty participants responded to PrEP modality preference questions (n = 69 HIV-negative individuals). Majority (76.8%) cited preference for oral agents, 13% intramuscular agents. Of 42 responses, 57.1% were not open to self-injection.

## Discussion

Our survey of individuals seeking mpox vaccination showed that the majority engaged in practices placing them at high risk for STIs, including HIV. These activities included not only sexual practices, but also other behaviors (such as illicit substance and alcohol use) that have been associated with increased HIV risk [13]. However, we found that almost half of these at-risk HIV-negative individuals were not on PrEP, and only a third had a recent HIV test (within 3 months).

In the US, there is a wide gap between PrEP need and use patterns, with only approximately 25% of PrEP-eligible individuals having received it [8]. As PrEP is one of the pillars of ending the U.S. HIV epidemic, these data are alarming. Thus, it is critical to enhance PrEP uptake and scale up effective evidence-based HIV prevention strategies (e.g., PrEP, HIV/STI testing, counseling) that decrease HIV incidence. In addition to promoting uptake, efforts should be made to support PrEP adherence, as our study and others have highlighted suboptimal PrEP adherence [14].

High-risk individuals for HIV may differ from the general population, in that they may be younger, be skewed towards racial and ethnic minorities, have lower socio-economic status, and more likely lack insurance [15]. Innovative approaches to engage such individuals are necessary, including community-based outreach and service delivery; use of non-traditional healthcare settings and telehealth may be quite effective. Another approach could offer comprehensive healthcare services to address multiple health needs in the same space and at the same time. In addition, high illicit substance and alcohol use rates suggest that opportunities exist to address HIV/STI prevention and substance use concurrently. Our data suggests that mpox vaccination centers may provide opportunities for PrEP services.

The majority of our survey respondents preferred oral PrEP formulations and were averse to self-injection. However, PrEP preference data is variable, as some studies have shown the reverse (with preference for injectables over oral tablets [16]). Regardless, there is a growing appetite for longer acting PrEP, which hold promise. To enhance uptake, future prevention interventions must include end-user input to ensure that needs and preferences are considered.

Our study has certain limitations. Our respondents’ demographics may differ from those of other locations, limiting external generalizability to those from dissimilar locations. We do note that it was a minority of individuals approached who completed the surveys. Thus, we acknowledge the internal and external generalizability may be impacted by that due to differing demographics. Individuals seeking mpox vaccination may be more risk-averse from a healthcare standpoint, have greater access to healthcare services, and thus be aware of and/or view HIV prevention favorably. While our self-administered questionnaire was anonymous, social desirability bias may have impacted self-reporting of HIV risk behaviors and substance use. However, the anonymity of the surveys could have overcome that limitation. In our study, we found high rates of self-reported substance use, and though not specifically assessed in our survey, may include chemsex which further exacerbates HIV risk behavior. Additionally, we did not pair questions on route of administration with dosing frequency, and it could have altered people’s preferences. For example, if a less desirable route of administration of PrEP is associated with less frequent dosing, it may be viewed more positively and impact reported preferences. Furthermore, not all individuals approached completed a survey, and those who did had not necessarily filled out all responses. Thus, this limits the total number of data points for certain questions (Additional file 1).

## Conclusion

Our cross-sectional survey highlights that individuals who were at risk for mpox infection and sought vaccination were also at risk for HIV infection. Furthermore, we found critical HIV prevention gaps and opportunities among individuals seeking mpox vaccination that could be addressed in the same context.

## Supplementary Information

**Additional file 1.** Anonymous survey—assessing HIV prevention preferences and needs of persons seeking Monkeypox vaccination.

## Data Availability

Data from this study are available but protected under the Yale Institutional Review Board given the sensitive nature of patient health information; therefore, restrictions apply to the availability of these data, which were used under license for the current study, and so are not publicly available. Data are however available from the authors upon reasonable request and with permission of Yale. The corresponding author can be contacted at Jessica.tuan@yale.edu.
