# Correction: HIV risk profile and prevention needs of individuals seeking monkeypox (mpox) vaccination in an urban clinic in the U.S.: a brief report

**DOI:** 10.1186/s12879-023-08159-2

**Published:** 2023-04-12

**Authors:** Onyema Ogbuagu, Manas Sharma, Grace Igiraneza, Laurie Andrews, Jessica Tuan, Lydia A. Barakat

**Affiliations:** 1 Section of Infectious Diseases, Yale School of Medicine, 135 College St., Suite 323, New Haven, CT 06510 USA; 2 Section of Infectious Diseases, Yale, Department of Medicine, Yale School of Medicine, Yale University School of Medicine, 333 Cedar Street, PO Box 208022, New Haven, CT 06510 USA; 3 Yale AIDS Program, 135 College St., Suite 323, New Haven, CT 06510 USA; 4 Yale AIDS Program, Section of Infectious Diseases, Yale School of Medicine, 135 College St., Suite 323, New Haven, CT 06510 USA

**Correction** **:** **BMC Infect Dis 23, 146 (2023)**

**https://doi.org/10.1186/s12879-023-08075-5**

Following publication of the original article [1], an error was identified in the Abstract, Results and Table 1 section.

**Table 1:** Demographics and Characteristics, History of At-Risk Behaviors, PrEP Awareness and Preferences of Survey Subjects

**Characteristics**	**n (%) or median (IQR)**	**HIV-positive** (*n* = 9)	**HIV-negative** (*n* = 69)
Race/Ethnicity	79 (100)	9 (100)	70 (100)
Black	13 (16.4)	2 (22.2)	11 (15.7)
White	48 (60.8)	4 (44.4)	44 (62.9)
Asian	14 (17.7)	2 (22.2)	12 (17.1)
Pacific Islander/Other	4 (5.1)	0 (0)	4 (5.7)
Mixed race	5 (6.3)	1 (11.1)	4 (5.7)
Hispanic/LatinX	12 (15.2)	1 (11.1)	11 (15.7)
Gender	81 (100)	9 (100)	68 (100)
Cis-Male	76 (93.8)	9 (100)	63 (92.6)
Cis-Female	2 (2.4)	0 (0)	2 (2.9)
Trans-male	1 (1.2)	0 (0)	1 (1.5)
Non-binary	1 (1.2)	0 (0)	1 (1.5)
Non-conforming	1 (1.2)	0 (0)	1 (1.5)
Age (years)	**28 (24–40)**	**39 (29–59)**	**27 (23–36.5)**
Occupation	77 (100)	9 (100)	68 (100)
Healthcare worker	10 (13)	1 (11.1)	10 (14.7)
Other at-risk occupation	6 (7.8)	0 (0)	6 (8.8)
Educational level	80 (100)	9 (100)	69 (100)
Less than high school	0 (0)	0 (0)	0 (0)
Completed high school	19 (23.8)	3 (33.3)	16 (23.2)
College degree	22 (27.5)	1 (11.1)	22 (31.9)
Post-graduate or other advanced degrees	36 (45)	5 (55.6)	30 (43.5)
Annual income	76 (100)	4 (100)	66 (100)
$75 K or greater	23 (30.3)	3 (75)	21 (31.8)
$40 K- $74 K	13 (17.1)	0 (0)	12 (18.2)
$20 K- $39 K	20 (26.3)	0 (0)	15 (22.7)
< $20 K	20 (26.3)	1 (25)	18 (27.3)
Medical Insurance	78 (100)	9 (100)	68 (100)
Private Insurance	64 (82.1)	5 (55.6)	57 (83.8)
Medicaid	7 (9)	2 (22.2)	5 (7.4)
Medicare	7 (9)	0 (0)	6 (8.8)
None	0 (0)		0 (0)
**Behaviors**			
Gender of sex partners	81 (100)	9 (100)	69 (100)
Male alone	69 (85.2)	8 (88.9)	58 (84.1)
Female alone	1 (1.2)	0 (0)	1 (1.4)
Both	11 (13.6)	1 (11.1)	10 (14.5)
Number of sexual partners			
Past 6 months	**4 (3–10)**	**4 (2–10)**	**4 (3–10)**
Past 2 weeks	**1 (1–2)**	**1 (1–1.5)**	**1 (1–2)**
Intimate behaviors/sexual practices	79 (100)	9 (100)	69 (100)
Hugging	70 (88.0)	5 (55.6)	64 (92.8)
Kissing	72 (91.1)	5 (55.6)	65 (94.2)
Oral intercourse	76 (96.2)	7 (77.8)	69 (100)
Insertive anal intercourse	55 (69.6)	5 (55.6)	48 (69.5)
Receptive anal intercourse	50 (63.3)	4 (44.4)	46 (66.7)
Insertive vaginal intercourse	6 (7.6)	1 (11.1)	5 (7.2)
Condom Usage	**90% (34.8–100%)**	**80% (25–100%)**	**90% (38.3–100%)**
HIV Status	81 (100)		
Positive	11.50%		
Negative	84.80%		
Unknown	3.70%		
STI history	81 (100)	9 (100)	69 (100)
Ever	33 (40.7)	7 (77.8)	25 (36.2)
Gonorrhea	17 (21)	5 (55.6)	12 (17.4)
Chlamydia	15 (18.5)	2 (22.2)	12 (17.4)
Syphilis	11 (13.6)	5 (55.56)	6 (8.7)
HPV	8 (9.9)	1 (11.1)	7 (10.1)
Genital herpes	1 (1.2)	1 (11.1)	0 (0)
Past 6 months	10 (12.3)	2 (22.22)	8 (9.9)
Gonorrhea	7 (8.6)	2 (22.2)	5 (6.2)
Chlamydia	3 (3.7)	2 (22.2)	2 (2.5)
Syphilis	2 (2.5)	1 (11.1)	2 (2.5)
Trichomoniasis	1 (1.2)	0 (0)	1 (1.2)
HPV	1 (1.2)	1 (11.1)	1 (1.2)
*M. genitalium*	1 (1.2)	0 (0)	1 (1.2)
Genital herpes	0 (0)	0 (0)	0 (0)
Substance use	77 (100)	9 (100)	67 (100)
No	34 (44.2)	3 (33.3)	31 (46.3)
Yes	43 (55.8)	6 (66.7)	36 (53.7)
Marijuana	37 (48.1)	4 (44.4)	32 (47.8)
Poppers or other inhalants	19 (24.7)	3 (33.3)	16 (23.9)
Cocaine	14 (18.2)	2 (22.2)	12 (17.9)
Ketamine	6 (7.8)	2 (22.2)	4 (6)
Methamphetamine	5 (6.5)	3 (33.3)	2 (3)
Mushrooms/psilocybin	3 (3.9)	0 (0)	3 (4.5)
MDMA	1 (1.3)	0 (0)	1 (1.5)
LSD	1 (1.3)	0 (0)	1 (1.5)
PCP	1 (1.3)	0 (0)	1 (1.5)
Heroin	1 (1.3)	0 (0)	1(1.5)
Tobacco	71 (100)	9 (100)	61 (100)
Yes	13 (18.3)	3 (33.3)	10 (13.1)
No	58 (81.7)	6 (66.7)	51 (86.9)
Alcohol	73 (100)	9 (100)	63 (100)
Yes	64 (87.7)	7 (77.8)	56 (88.9)
Moderate	64 (87.7)	7 (100)	56 (88.9)
Heavy	0 (0)	0 (0)	0 (0)
No	9 (12.3)	2 (22.2)	7 (11.1)
**Behaviors**			
Aware of PrEP	79 (100)	9 (100)	69 (100)
Yes	75 (94.9)	8 (88.9)	66 (95.7)
No	4 (5.1)	1 (11.1)	3 (4.3)
Brand Awareness	65 (100)	2 (100)	63 (100)
Tenofovir disoproxil fumarate/	43 (65.8)	2 (100)	41 (65.1)
emtricitabine	29 (44.4)	2 (100)	27 (42.9)
Tenofovir alafenamide/	4 (6.3)	0 (0)	4 (6.3)
emtricitabine			
Cabotegravir			
PrEP Usage	69 (100)		69 (100)
Yes	33 (48.1)		33 (48.1)
No	36 (51.9)		36 (51.9)
Brand Usage	33 (100)		33 (100)
Tenofovir disoproxil fumarate/emtricitabine	21 (63.6)		21 (63.6)
Tenofovir alafenamide/emtricitabine	11 (33.3)		11 (33.3)
Cabotegravir	1 (3)		1 (3)
Prescription Provider	32 (100)		32 (100)
Primary Care Provider	24 (75)		24 (75)
HIV Specialist/Clinician	4 (12.5)		4 (12.5)
Other sources	4 (12.5)		4 (12.5)
Adherence	28 (100)		28 (100)
Full (no missed doses)	21 (75)		21 (75)
At least 4 doses per week	2 (7)		2 (7)
Less than 4 doses a week	5 (17.9)		5 (17.9)
Preferred Route of Administration	40 (100)		40 (100)
Oral agent	30 (75.8)		30 (75.8)
Injectable agent into muscle	5 (13)		5 (13)
Injectable agent into skin	4 (10.1)		4 (10.1)
Implant	1 (4.3)		1 (4.3)
Openness to Self-Injection	42 (100)		42 (100)
Yes	18 (42.9)		18 (42.9)
No	24 (57.1)		24 (57.1)
Preferred Frequency	44 (100)		44 (100)
Daily	7 (15.9)		7 (15.9)
Weekly	11 (25)		11 (25)
Monthly	14 (31.8)		14 (31.8)
Every 2 months	5 (11.2)		5 (11.2)
Every 6 months	8 (18.4)		8 (18.4)
1 year or more	17 (38.6)		17 (38.6)

The updated sections are provided below and the changes have been highlighted in **bold typeface**.

**Abstract**

“Eighty-one of 210 individuals approached completed surveys (survey acceptance and completion rate 38.6%). Majority were cisgender-male (76/81; 93.8%), Caucasian (48/79; 60.8%), with median age 28 years **(IQR 24-40)**. Nine of 81 (11.5%) self-reported HIV-positivity. Median sexual partner number (6 months prior) was 4 **(IQR 3-10)**. Majority, 89.9% and 75.9%, reported insertive and receptive anal intercourse, respectively. 41% reported lifetime STI history, of whom 12.3% had an STI 6 months prior. Majority (55.8%) used ≥ 1 illicit substance; 87.7% moderate alcohol use. Among HIV-negative respondents, most (95.7%) were aware of PrEP, but only 48.4% used PrEP.”

**Results**

“The gender of sexual partners was only male for 85.2%, while 13.6% reported both, and 1.2% selected only female partners. Median values for number of sexual partners in prior 6 months and 2 weeks were 4 **(IQR 3-10)** and 1 **(IQR 1-2)**, respectively. 12.3% had partners living with HIV (Table 1). Regarding sexual practices, 96.2% participated in oral sex, 69.6% insertive anal intercourse, 63.3% receptive anal intercourse, and 7.6% insertive vaginal intercourse. Median percentage of condom usage was 90% **(IQR 34.8-100%)**. Majority (94.9%) reported no known mpox exposure, while 3.8% reported known exposure.”

Following publication of the original article [1], the authors identified an error in Table 1. The correct table is given below.

The original article [1] has been corrected.
