# Supplementary material for: HIV risk profile and prevention needs of individuals seeking monkeypox (mpox) vaccination in an urban clinic in the U.S.: a brief report
Source: BMC Infect Dis. 2023 Mar 8;23:146. doi: 10.1186/s12879-023-08075-5 (PMC9993362; doi:10.1186/s12879-023-08075-5)
Supplement: Supplementary file 1 — Additional file 1. Anonymous survey—assessing HIV prevention preferences and needs of persons seeking Monkeypox vaccination. [file 12879_2023_8075_MOESM1_ESM.docx]

**Anonymous Survey
Assessing HIV prevention preferences and needs of persons seeking Monkeypox vaccination**

**Demographics:**

1. Gender at birth: ] Male [] Female
2. Gender now: [] Male [] Female [] other non-binary _____________
3. Age ___________ years
4. Occupation: [] healthcare worker [] other job that put you at risk for monkeypox such as laboratory work, animal handler) [] not a healthcare worker
5. Race: [] Black [] White [] Asian [] American Indian/ Alaskan Native [] Native Hawaiian [] Other pacific Islander
6. Ethnicity: [] Hispanic [] Non-Hispanic
7. Educational level (list highest completed level) [] less than high school [] high school [] college [] postgraduate or advanced degree
8. Estimated annual Income: [] <20k [] 20-39K [] 40-74K [] 75K or more
9. Insurance: [] Private [] Medicaid [] Medicare [] uninsured
10. Marital Status: [] married (monogamous) [] married (non-monogamous) [] single [] co-habiting [] divorced

**Sexual History**

1. Gender of sexual partners [] male [] female [] both
2. Number of sexual partners in past 6 months _____________
3. Number of sexual partners in past 2 weeks _________________
4. What are the range of your intimate behaviors and your sexual practices – (choose all that apply) [] hugging [] kissing [] oral sex [] receptive anal intercourse [] insertive anal intercourse [] insertive vaginal intercourse
5. What percentage of times during intercourse do you wear a condom (choose a number from 0 to 100%)

0______________________________________________100%

1. Are any of your sexual partners living with HIV (HIV positive) [] yes []no
2. Have you been exposed to someone with monkeypox in past 2 weeks?

[] yes [] no

**History of STDs**

1. Have you **ever** had:

[] gonorrhea [] syphilis [] chlamydia [] trichomonas [] Mycoplasma genitalium

[] other non- gonococcal urethritis [] HPV [] genital herpes

1. In the past 6 months have you been diagnosed with and/or treated for :

[] gonorrhea [] syphilis [] chlamydia [] trichomonas [] Mycoplasma genitalium

[] other non- gonococcal urethritis [] HPV [] genital herpes

1. Have you ever tested positive for HIV []yes []no
2. If no, when was your last KNOWN negative HIV test ____________ (date)

**Substance Use**

1. Have you ever used any illicit substances? [] Yes [] No
2. If Yes, which one (s)

[] metamphetamines [] poppers [] heroin [] cocaine [] ketamine [] PCP [] marijuana [] other ____________

1. Have you ever used drugs by injection? [] yes [] no
2. If you use injection drugs- do you ever share needles or injection equipment? [] Yes [] No
3. Do you smoke tobacco: quantify daily use _____________
4. Do you drink alcohol: quantify weekly use ______________

**PrEP awareness and preference**

1. Have you heard of HIV Pre-exposure prophylaxis (PrEP)? [] Yes [] No
2. If NO, would you like us to learn more about it? [] Yes [] NO (if Yes, please notify the front desk of your interest) then **skip the rest of this section.**
3. If Yes to #29 (you are aware of PrEP), are you using PrEP? [] Yes []NO
4. What kind of PrEP have you heard about (Choose all that you know) [] Truvada [] Descovy [] Cabotegravir (Apretude)
5. What kind of PrEP are you using? [] Truvada [] Descovy [] Cabotegravir (Apretude)
6. Who is providing you with PrEP [] primary care provider [] HIV specialist or clinic [] planned parenthood [] community healthcare van [] other (specify)____________
7. IF using a form of oral PrEP, rate the level of your adherence [] full (no missed doses in past month) [] partial [] at least 4 doses a week [] less than 4 doses per week (i.e. more than 3 missed doses per week)
8. Are you happy with the form of PrEP you are using? [] Yes [] No. If NO, Why? ______________________
9. If available, What are your optimal preferences for HIV PrEP (choose one)

[] oral agent [] injectable agent (into muscle) [] injectable agent [into skin]

[] implant

1. are you open to self-injections for PrEP [] yes [] no
2. If available, What would be your optimal frequency for use of a PrEP agent (Choose one)

[] daily [] weekly [] monthly [] every 2 months [] every 6 months [] 1 year or more
